# Traumatic lens dislocation

**DOI:** 10.1186/s12245-015-0064-5

**Published:** 2015-05-27

**Authors:** Sangil Lee, Alison Hayward, Venkatesh R Bellamkonda

**Affiliations:** Department of Emergency Medicine, Mayo Clinic Health System, 1453 Marsh Street, Mankato, MN 56001 USA; Department of Emergency Medicine, Yale University, 464 Congress Ave, Ste 260, New Haven, CT 06519-1315 USA; Department of Emergency Medicine, Mayo Clinic, 200 2nd Street, Rochester, MN 55901 USA

**Keywords:** Point-of-care ultrasound, Lens dislocation, Ocular trauma

## Abstract

**Background:**

Point-of-care ultrasound has been widely used by clinicians, particularly those in emergency care settings.

**Findings:**

A 44-year-old male who sustained a blunt ocular trauma resulting in acute vision loss due to posterior dislocation of the crystalline lens identified by point-of-care ultrasound is described in the study. Point-of-care ultrasound with a high linear-array transducer was used to gain the sagittal view of the eye globe.

**Conclusions:**

Point-of-care ultrasound can be a useful tool to make a rapid diagnosis of ocular emergency including lens abnormality.

## Findings

### Case synopsis

**Fig. 1:**
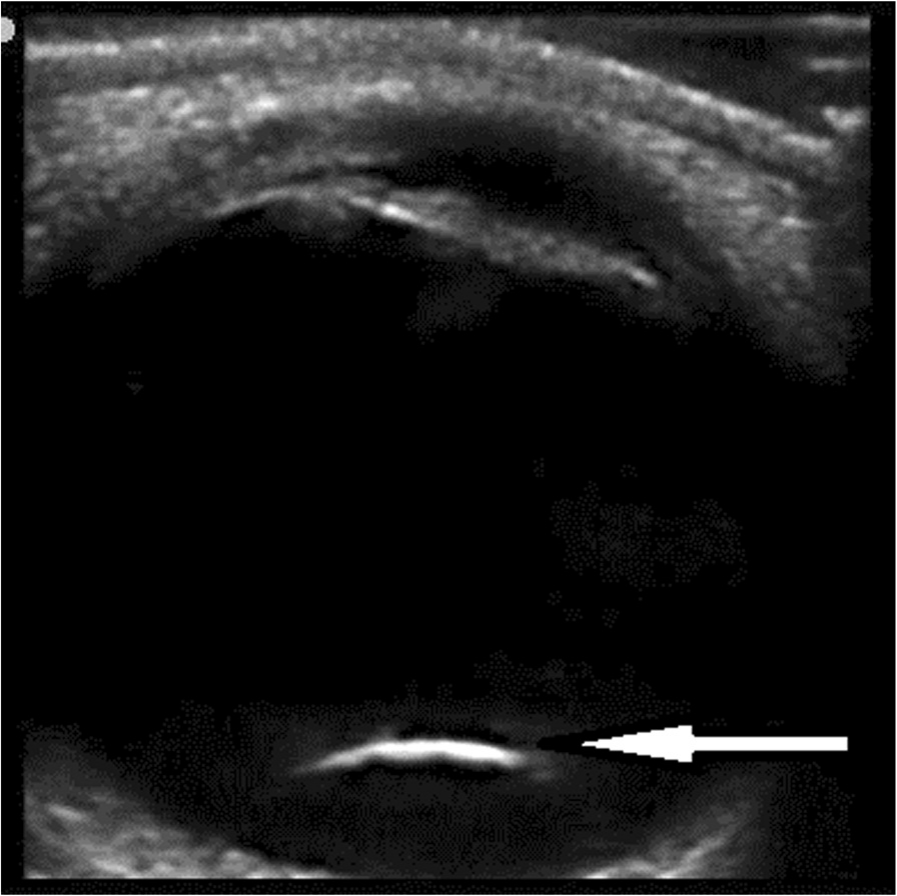
Sagittal view of left eye globe showing floating lens in the posterior chamber (*white arrow*)

**Fig. 2:**
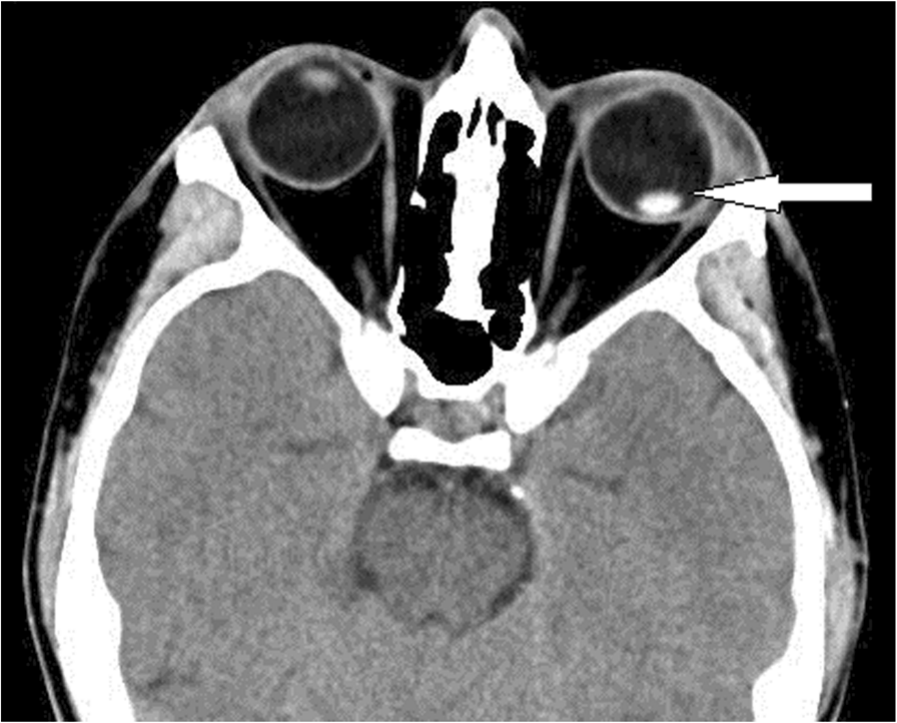
CT image corresponding to point-of-care ultrasound showing dislocated lens (*white arrow*)

A 44-year-old man was fixing his mountain bike when the shock absorber broke apart, shooting a cap which struck him in the left eye. Intense pain and vision loss developed. Initial vital signs were stable (BP 117/79 mmHg, pulse 80 bpm, respiratory rate 16 breaths per minute, oxygen saturation 99 % on room air). Physical exam revealed that the patient had a laceration to his superior eyelid, a subconjunctival hemorrhage, and a hyphema. The deformity of the left pupil was noticed. He was only able to perceive light from the affected eye. Head and neck exams were otherwise unremarkable. After topical anesthetics were given, a 13-6-MHz linear-array ultrasound probe was applied using a closed-eye technique. Point-of-care ultrasound and CT imaging of the patient are shown in Figs. 1 and 2. CT showed no skull fracture, facial fracture, or intracranial abnormality. Diagnosis of traumatic lens dislocation was confirmed. This patient was reimmunized against tetanus, given antibiotics, provided an eye shield, and then taken to the operating room for management by ophthalmology. Exploration showed posterior lens dislocation, upper lid laceration without globe rupture. Postoperatively, intraocular pressure rose to 38 mmHg and Timolol, Brimonidine were started. The patient was released on postoperative day three. A consent was obtained from the patient.

### Discussion

Point-of-care ultrasound demonstrates a displaced lens in the posterior chamber (Fig. 1), which is confirmed by CT scan (Fig. 2). Lens dislocation is a rare complication of head injuries. However, trauma is the most common cause of lens dislocation [1]. Blunt force in anteroposterior direction leads to equatorial expansion, which disrupts the zonular fibers and dislocates the lens [2]. Connective tissue disorders and primary ocular disorders should be considered in the absence of trauma. Patients with Marfan’s syndrome have better prognosis than those with traumatic lens displacement; however, for both conditions, the displacement of the lens is a threat to vision [1].

Point-of-care ultrasound can quickly establish the diagnosis and prompt ophthalmology consultation without CT results. Ultrasound is used primarily to evaluate internal structures of the globe, especially when direct visualization is difficult due to cataracts or hemorrhage. It can help to detect choroidal or retinal detachment, and also has some retro-ocular applications. A closed-eye technique is performed by placing a high linear-array ultrasound transducer to the patient’s closed eyelid. A large amount of water soluble gel is used to avoid direct contact with the eyelid. The globe should be evaluated in sagittal and transverse planes. The dilatation of pupils and the use of ophthalmoscope can be obviated by point-of-care ultrasound.

The complications of lens dislocation include secondary glaucoma, retinal detachment, cataract, and vision loss [3]. Surgical repair is often the treatment of choice, including repositioning, explanting, or exchanging the displaced intraocular lens particularly when dislocation is a result of trauma [4]. Our patient developed secondary glaucoma, proliferative vitreoretinopathy and required lens removal.

#### Teaching pearls

1. Traumatic lens dislocation is one of the differentials for irregular pupil after trauma.
2. Point-of-care ultrasound is an excellent diagnostic modality for eye pathology.
3. Contraindication for ultrasound is globe rupture.

## Abbreviation

CT: Computed tomography
